# Deep Q-Network-Based Cooperative Transmission Joint Strategy Optimization Algorithm for Energy Harvesting-Powered Underwater Acoustic Sensor Networks

**DOI:** 10.3390/s20226519

**Published:** 2020-11-14

**Authors:** Song Han, Luo Li, Xinbin Li

**Affiliations:** Institute of Electrical Engineering, Yanshan University, Qinhuangdao 066004, China; liluo630@163.com (L.L.); lixb@ysu.edu.cn (X.L.)

**Keywords:** underwater acoustic sensor networks, cooperative transmission, joint relay selection and power allocation, energy harvesting, reinforcement learning

## Abstract

Cooperative transmission is a promising technology for underwater acoustic sensor networks (UASNs) to ensure the effective collection of underwater information. In this paper, we study the joint relay selection and power allocation problem to maximize the cumulative quality of information transmission in energy harvesting-powered UASNs (EH-UASNs). First, we formulate the process of cooperative transmission with joint strategy optimization as a Markov decision process model. In the proposed model, an effective state expression is presented to better reveal interactive relationship between learning and environment, thereby improving the learning ability. Then, we further propose a novel reward function which can guide nodes to adjust power strategy adaptively to balance instantaneous capacity and long-term quality of service (QoS) under the dynamic unpredictable energy harvesting. More specifically, we propose a deep Q-network-based resource allocation algorithm for EH-UASNs to solve the complex coupled strategy optimization problem without any prior underwater environment information. Finally, simulation results verify the superior performance of the proposed algorithm in improving the cumulative network capacity and reducing outages.

## 1. Introduction

Recently, underwater acoustic sensor networks (UASNs) have attracted widespread research attention due to the emerging applications in various marine fields [1,2]. For UASNs, it is important to transmit effectively the crucial information which is captured by sensors in the complex underwater environment. Given that underwater acoustic communication has the characteristics of limited energy, severe fading, time-varying channel and high transmission delay, it is a challenge to guarantee the large information transmission range and high quality of service (QoS) in UASNs [3,4]. The relay-assisted UASNs can achieve effective information transmission in a long range by deploying relay sensor nodes. Therefore, in energy constrained relay-assisted UASNs, cooperative transmission joint sub-strategies (relay selection and power allocation) optimization algorithms have become a key technology to improve the network performances [5,6,7].

Scholars have done a mass of researches on resource allocation to improve the performance of UASNs. In Reference [8], Wei et al. presented a cooperative relay scheme to improve the reliability of information transmission in UASNs under the fixed transmitting power. In this scheme, the “best” relay was selected from a set of potential neighbor nodes based on the distance and perfect instantaneous channel conditions. In Reference [9], Tran et al. proposed a routing algorithm to select routing relays and cooperative relays. The best relay which can maximize Signal to Noise Ratio (SNR) would be selected to forward information with constant transmitting power. All the aforementioned algorithms assumed the perfect prior channel state information (CSI) was known. However, owing to the severe propagation delay in the complex underwater environment, the real-time perfect CSI is hard to obtain [10]. Therefore, the assumption cannot be easily satisfied in UASNs. If the imprecise CSI is employed in the above algorithms, the performance will degenerate seriously. In addition, in the above literatures, only the relay selection strategy was optimized with the constant transmitting power. Although the above optimization problems are easy to solve, the joint optimization approaches of relay selection and power allocation can provide more abundant and effective means to improve energy efficiency and QoS, especially in energy constrained relay-assisted UASNs.

The joint relay selection and power allocation can more effectively maximize the network potential performance and QoS. In Reference [11], Tian et al. proposed a joint relay selection and resource allocation scheme to maximize the total transmission data rates for the D2D network. In this scheme, a social-aware relay selection algorithm was first proposed to select relay. And then, the optimal power was allocated to maximize the transmission data rates based on perfect CSI. Given that the harsh underwater environment, the algorithm cannot be directly applied to UASNs. In Reference [12], Doosti et al. proposed an adaptive relay selection and power allocation algorithm for UASNs to improve capacity and reduce bit error rate based on perfect CSI. In this algorithm, the optimal relay was first selected, and then the optimal power allocation problem was solved. For the underwater optical wireless communication [13], the relay assignment sub-problem was solved by an improved Kuhn-Munkres algorithm and power allocation sub-problem was solved by an iterative bisection algorithm. Although the aforementioned literatures improved the instantaneous QoS, the joint resource allocation problem was solved by decomposing it into two sub-problems. They neglected the coupling of relay selection and power allocation to easy the difficulty of solving, which may lead to performance degradation. Moreover, the energy efficiency was ignored in the above literatures. It may cause overuse of energy, thereby reducing working life and long-term QoS. Consequently, the aforementioned joint resource allocation algorithms cannot be directly applied to energy-constrained UASNs.

As we all know, if the nodes transmit signals with higher power, the network will obtain higher capacity. Given that limited energy of nodes in UASNs, if the nodes keep working with high transmitting power, the batteries need to be replaced frequently to maintain long-term service. However, due to the particularity of underwater operations, it is impossible to afford the high cost of replacing batteries. Energy Harvesting (EH) technology is a promising solution to reduce battery replacement and maintain the working life of nodes. Nodes with EH capabilities can exploit harvested energy from the marine environment to power themselves through rechargeable batteries [14]. Based on a fixed harvested energy conversion efficiency of EH [15], Song et al. proposed a joint optimal power allocation and relay selection strategy to maximize the system information rate of relay network. In addition, the joint optimization problem was decomposed into two sub-problems to address in two phases. Owing to the stochastic and intermittent nature of EH process, the remaining energy of nodes is dynamic and unpredictable. Therefore, the solving of joint resource allocation problem is more difficult. Under the dynamic energy harvesting [16], Jiao et al. developed a novel evolutionary algorithm to jointly optimize the data rates and power allocation to decrease the total network delay for wireless sensor networks. In Reference [17], Jing et al. employed a channel-aware dynamic programming approach to allocate power to maximize capacity of EH-UASNs. In this approach, the authors concentrated on the distribution of battery energy for sensing and transmission. Moreover, the balance between remaining energy and energy efficiency was not considered in the above literatures. Therefore, the benefits of EH technology are not fully exploited to prolong working life of nodes.

In face of harsh underwater environment including unknown CSI and dynamic EH, traditional optimization algorithms cannot directly solve dynamic joint optimization problem which includes unknown factors. Reinforcement learning (RL) based on artificial intelligence has been used as a powerful tool to solve complex resource allocation problem in various wireless networks with poor prior information [18,19,20]. In Reference [21], Running et al. proposed a model-based RL power allocation algorithm for the energy harvesting underwater relay networks to maximize the long-term end-to-end information transmission rate. And in this algorithm, it assumed channel gain information is invariant for a while. However, the acoustic channels are strongly time-varying in underwater. For time-varying channel environment [22], Hui et al. proposed a Q-learning-based power allocation to alleviate interference in underwater communication, thereby improving the networks capacity. In Reference [23], Valerio et al. proposed a Channel-aware Q-learning-based Multi-path Adaptive routing algorithm to optimize route-long energy consumption and packet delivery ratio for time-varying underwater channel environment. The above RL-based resource allocation algorithms made decisions, based on a finite Q-table, which map the relationship between the state space and the action space. In the practice, the underwater environment is complex and dynamic, which will cause the Q-table to be very large. At the same time, storage space and search time will be increased sharply. Generally, the state space is discretized or state transition probability is known to reduce the dimension of the Q-table. However, it is hard to model the underwater acoustic channel accurately. Therefore, the above algorithms cannot be directly applied to UASNs. Otherwise, the algorithm performance will be seriously degraded. Therefore, the main pain point is how to deal with the infinite state space without modeling underwater channel. Deep reinforcement learning (DRL), which introduces neural networks into reinforcement learning, was proposed by Deepmind to cope with complex problems. The DRL algorithms are not limited by the size of the state space, and can directly use the high-dimensional state training to help the agent learn effective strategies. Especially when solving the complex decision-making problems, the DRL algorithms are proved to be competent for challenging tasks, such as Go and Atari games [24,25,26]. Scholars have employed the advantages of DRL to optimize the performance of wireless networks. In Reference [27], a DRL-based relay selection algorithm in WSNs was presented, in which mutual information and instantaneous SNR were utilized as state information to learn relay selection strategy. However, in this algorithm, the channel gain information was discretized and its transition probability was known. Although the relevant performance evaluation indicated that the proposed scheme was superior to the Q-learning-based scheme, the algorithm [27] cannot be directly applied to UASNs. For the UASNs [28], Xiao et al. proposed a DRL-based antijamming relay scheme to reduce bit error rate and improve the utility of the relay node. Based on the perfect real-time CSI, the relay decided whether to leave the heavily jammed location to mitigate interference. However, this scheme did not fully consider the characteristics of time-varying channel and high transmission delay in underwater environment, as well as the service requirements of long-term deployment. An overview of the existing studies for the underwater networks mentioned above are listed in Table 1.

In this study, we study the joint relay selection and power allocation problem to maximize the cumulative quality of information transmission and model the process of cooperative transmission with joint strategy optimization as a Markov decision process in EH-UANSs. We propose a joint resource allocation based deep Q network algorithm considering energy harvesting (JADQ-EH) to optimize the performance of EH-UASNs. In the proposed algorithm, we fully consider uncertainty of the underwater environment, the hysteresis of channel information caused by transmission delay and the dynamic stochasticity of EH. We first propose an effective state expression which does not require any prior channel information to cope with the harsh underwater environment. The state expression which includes outdated channel information, topological information and nodes energy state information can provide available abundant learning information, thereby improving learning ability. A novel reward function is further proposed to adaptively utilize harvested energy and improve the resource utilization by comprehensively considering harvested energy and instantaneous transmitting power of relay node. The proposed JADQ-EH algorithm can not only reduce outage probability but also balance the instantaneous capacity and long-term QoS.

The main contributions of this paper are as follows:

1. We develop a cooperative transmission pattern in UASNs and formulate the process of cooperative transmission with joint strategy optimization as a Markov decision process (MDP) without the knowledge of the cooperative transmission model. Considering the limited energy of nodes, the energy harvesting technology is applied to improve long-term QoS. In addition, we present herein the practical analysis of joint relay selection and power allocation in EH-UASNs.
2. We propose the JADQ-EH algorithm based on deep Q network to learn the joint optimization strategy for EH-UASNs. An effective state expression which does not require any prior channel information is proposed to provide available abundant learning information for the proposed algorithm, thereby improving learning ability. The proposed algorithm does not need to discretize channel information and can directly use raw data obtained from underwater environment to train. Therefore, the proposed learning algorithm is highly applicable to dynamic unknown UASNs.
3. Given that the dynamic stochastic nature of energy harvesting, we propose a novel reward function to utilize harvested energy adaptively. The proposed reward function can balance instantaneous capacity performance and long-term working life of EH-UASNs. With the guidance of reward function, the proposed JADQ-EH algorithm can increase the achievable cumulative capacity while minimizing the outage probability.

The rest of this study is organized as follows. Section 2 describes the model of EH-UASNs. In Section 3, the joint relay selection and power allocation problem formulation is presented. And the details of the proposed JADQ-EH algorithm and the corresponding analysis are shown in this Section. Simulation results are shown to validate the analysis in Section 4. Finally, we summarize this study in Section 5.

## 2. System Model

### 2.1. Network Model

**Figure 1:**
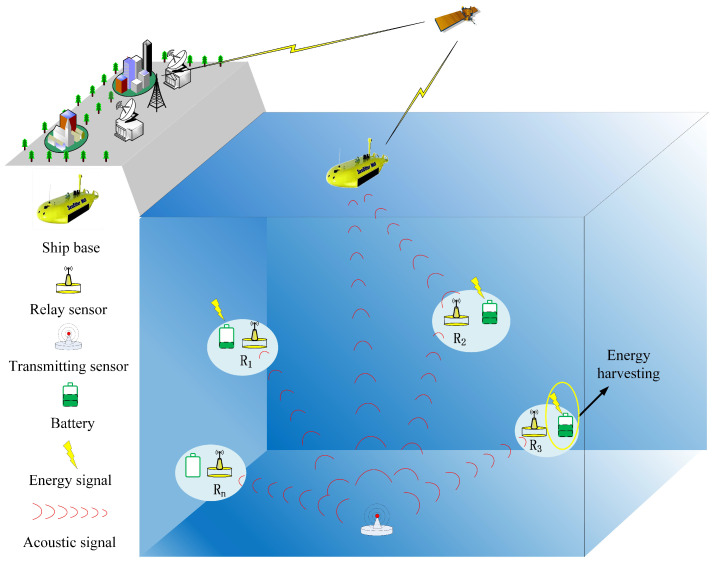
Illustration of underwater acoustic sensor networks (UASNs) considering energy harvesting.

In this study, we consider an UASN as illustrated in Figure 1. In the UASN, several underwater sensors are deployed underwater for a long time to sense important underwater information. These sensor nodes are equipped with underwater acoustic communication function to transmit the collected expected underwater data. The expected underwater data should be transmitted between sensor nodes and ship base in underwater environment. And then, the important information can be obtained by land base station. Given that the underwater acoustic channel attenuation is severe and the sensor nodes cannot be equipped with a high-power transmitter, the collected data is usually forwarded through relay to improve information transmission reliability and QoS [29]. Following this relay idea, we consider an UASN consists of a transmitting node *S*, a receiving node *D*, and *N* half-duplex relay nodes $R_{n},n\in N=\left\{1,...,N\right\}$ which are randomly distributed between *S* and *D*. The desired signal can reach *D* directly from *S*, and it can also be forwarded to *D* by a relay node. It is worth noting that the data transmission is time slotted and in each time slot, the transmitting node *S* can only be served by one relay node [27].

Let $g_{SR_{n}}^{k}$, $g_{R_{n}D}^{k}$ and $g_{SD}^{k}$ denote the channel gain from *S* to node $R_{n}$, the channel gain from $R_{n}$ to *D* and the channel gain from *S* to *D* in time slot *k*, respectively. The channel gain $g_{i,j}^{k}$ between node *i* and node *j* in time slot *k* is expressed as:

(1) $$\begin{array}{c} g_{i,j}^{k}=h^{k}A\left(d_{i,j},f\right), \end{array}$$

(2) $$\begin{array}{c} A\left(d_{i,j},f\right)=d_{i,j}^{\delta }a{\left(f\right)}^{d_{i,j}}, \end{array}$$

(3) $$\begin{array}{c} a\left(f\right)=10^{0.011\frac{f^{2}}{1+f^{2}}+4.4\frac{f^{2}}{4100+f^{2}}+2.75\times 10^{-5}f^{2}+3\times 10^{-4}}, \end{array}$$

where $h^{k}$ is the channel fading in time slot *k*. The *h* obeys the Rayleigh distribution. $A(d_{i,j},f)$ is channel path loss. $d_{i,j}$ is the distance between node *i* and node *j*. δ is the expansion coefficient which is taken to be 1.5. a(f) is absorption coefficient that can be expressed by the Thorp empirical formula [30]. *f* is the communication frequency.

The SNR obtained by the relay sensor $R_{n}$ in time slot *k* can be expressed as:

(4) $$\begin{array}{c} \Gamma_{SR_{n}}^{k}=\frac{P_{S}g_{SR_{n}}^{k}}{\sigma^{2}}, \end{array}$$

and the SNR obtained by *D* in time slot *k* can be expressed as:

(5) $$\begin{array}{c} \Gamma_{SD}^{k}=\frac{P_{S}g_{SD}^{k}}{\sigma^{2}}, \end{array}$$

the SNR from the relay node $R_{n}$ obtained by *D* in time slot *k* can be expressed as:

(6) $$\begin{array}{c} \Gamma_{RD}^{k}=\frac{P_{R_{n}}^{k}g_{R_{n}D}^{k}}{\sigma^{2}}, \end{array}$$

where $P_{S}$ and $P_{R_{n}}^{k}$ are the transmitting powers of *S* and relay node $R_{n}$, respectively. In addition, $\sigma^{2}$ donates the noise, which consists of turbulence, shipping, waves, and thermal noise [31].

(7) $$\begin{array}{c} 10\log N_{t}\left(f\right)=17-30\log\left(f\right); \end{array}$$

(8) $$\begin{array}{c} 10\log N_{\mu }\left(f\right)=40+20\left(\mu -0.5\right)+26\log\left(f\right)-60\log\left(f+0.03\right); \end{array}$$

(9) $$\begin{array}{c} 10\log N_{w}\left(f\right)=50+7.5w^{1/2}+20\log\left(f\right)-40\log\left(f+0.4\right); \end{array}$$

(10) $$\begin{array}{c} 10\log N_{th}\left(f\right)=-15+20\log\left(f\right); \end{array}$$

(11) $$\begin{array}{c} N\left(f\right)=N_{t}\left(f\right)+N_{\mu }\left(f\right)+N_{w}\left(f\right)+N_{th}\left(f\right). \end{array}$$

(7)–(10) indicate the impacts of these four aspects, respectively. Thus, the overall power spectral densities of the underwater ambient noise can be expressed as (11). The μ∈[0,1] indicates the impact of transport, and *w* is the wind speed.

Therefore, the capacity of the entire sensor network at the end of time slot *k* is

(12) $$\begin{array}{c} C^{k}=\frac{1}{2}\Phi \log_{2}\left(1+\Gamma_{SD}^{k}+\frac{\Gamma_{SR_{n}}^{k}\Gamma_{RD}^{k}}{1+\Gamma_{SR_{n}}^{k}+\Gamma_{RD}^{k}}\right), \end{array}$$

where Φ is the channel bandwidth.

### 2.2. Energy Harvesting Model

Given that the working life and transmitting power are restricted by the limited battery of relay nodes, the EH technology has been employed to improve the QoS and extend the lifetime of relay nodes in EH-UASNs [14]. The relay nodes can convert the available energy in the marine environment into rechargeable batteries to power themselves. In any time slot *k*, relay nodes can harvest energy and forward signals simultaneously. The harvested energy in time slot k−1 is collected and stored in a battery to support follow-up work. The maximum battery capacity of relay node $R_{n}$, n∈N is $B_{max}$. We assume that the EH process is an independent uniform distribution in [0, 3] mJ. It is worth noting that the battery of relay nodes at the beginning of time slot *k* $B_{n}^{k}=min\left\{B_{max},B_{n}^{k-1}+E_{n}^{k-1}-e_{n}^{k-1}\right\}$. Let $E_{n}^{k-1}$ donate the harvested energy by relay node $R_{n}$ in time slot k−1, and $e_{n}^{k-1}$ donate the energy consumed by the relay task in time slot k−1. Without loss of generality, it is reasonable to assume that the initial battery $B_{n}^{0}=B_{max}$, n∈N.

## 3. Problem Formulation and Solution

### 3.1. Problem Formulation

In this study, our goal is to maximize the continuous working cumulative capacity of the EH-UASNs by optimizing joint relay selection and power allocation. As we all know, the capacity obtained by node *D* increases as transmitting power of relay node increases. However, given that the energy of underwater sensors is limited, if the transmitting power is blindly increased to improve network capacity, it may cause frequent outages due to energy exhaustion, thereby strongly reducing the working life of nodes and QoS of networks. Therefore, the performance of UASNs will be seriously degraded.

Based on the analysis, the optimization problem of joint relay selection and power allocation for EH-UASNs in continuous *K* time slots, a working period, can be described as:

(13) $$\begin{array}{c} \max_{n,P_{R_{n}}}\sum_{k=1}^{K}C^{k}, \end{array}$$

(14) $$\begin{array}{c} s.t.\;\;\;P_{R_{n}}^{k}\leq P_{n,max},\;\forall k\in \left\{1,2,...,K\right\},\;n\in N, \end{array}$$

(15) $$\begin{array}{c} \;e_{n}^{k}\leq B_{n}^{k},\;\forall k\in \left\{1,2,...,K\right\},\;n\in N, \end{array}$$

where $P_{n,max}$ is maximum transmitting power of relay $R_{n}$, n∈N. It is worth noting that each relay node has a certain working range of transmission power. (14) is the transmission power constraint of each relay node $R_{n}$, n∈N at the time slot *k*. It means that the allocated power of the selected relay node should always not more than its working range of transmission power at the time slot *k*. (15) denotes energy consumed constraint of relay $R_{n}$, n∈N at the time slot *k*. On the other words, the consumed energy of the selected relay node $R_{n}$, n∈N should always be not more than the available battery energy $B_{n}^{k}$ at the time slot *k*. Otherwise, corresponding link will produce an outage.

Different from References [21,27], we consider a complex joint relay selection and power allocation problem for EH-UASNs, rather than the single power allocation or relay selection. In addition, the traditional relay network optimization algorithms [8,9] assume the perfect real-time CSI is known. However, it is infeasible in dynamic underwater environment. In this study, we do not require any prior environment information. Therefore, the applicability of our study is strong. In addition, the optimization problem of maximizing cumulative capacity in a working period ($C_{total}$) is formulated under the dual dynamic characteristics of the external time-varying channel environment and the internal unpredictable remaining energy. Although the proposed optimization problem reveals the operational characteristics of EH-UANSs well, it is more difficult to solve. The traditional algorithms can only optimize the current instantaneous performance based on past and current knowledge of system state. They cannot maximize the future cumulative performance without future information. In this study, the long-term future cumulative performance is our optimization objective. Moreover, only the current information of unpredictable EH can be obtained, not the future information. Therefore, the traditional algorithms are harder to find the global optimal solution to optimize the cumulative performance in a working period.

### 3.2. RL-Based EH-Uasns Joint Resources Allocation Algorithm

Machine learning can solve the complex optimization problem by learning without prior information. As an effective machine learning method which can maximize both current and expected future performance, the RL algorithm allows the agent to continuously interact with the dynamic environment and learn the revenue contribution of the current decision in the entire working period. In order to maximize the benefits of the entire working period, a part of the current benefits may be temporarily sacrificed. Inspired by the above analysis, a joint relay selection and power allocation algorithm framework based on RL is proposed to solve the complex coupled problem in a highly dynamic unknown underwater environment.

Based on the formulated optimization problem, we assume that the relay selection and power allocation process for EH-UASNs is a state transition process. The state in the (k+1)th time slot is only related with the state and action in the *k*th time slot. It is actually a MDP [27]. The state changes discontinuously when an action is performed.

**Figure 2:**
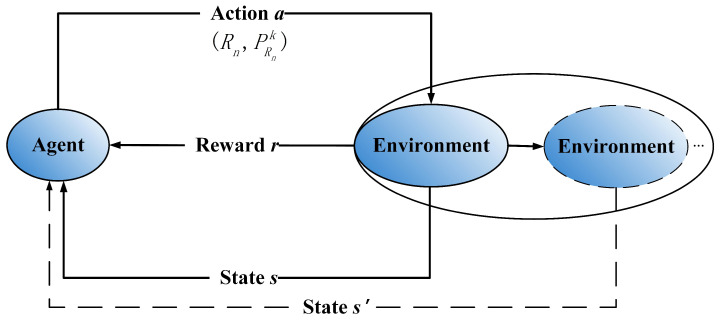
Framework of reinforcement learning.

Based on the given analysis, RL is employed to solve joint relay selection and power allocation problem for EH-UASNs which is a MDP model. First, the basic framework of RL is briefly introduced to show the proposed algorithm clearly. In Figure 2, the agent selects an action *a* according to the system state *s* and executes the action *a*. Then, the environment transfers from state *s* to next state $s^{\prime }$ and the agent obtains a reward *r*. In this way, the agent interacts with the environment continuously and adjusts its action strategy according to the reward *r*. In this study, the transmitting node *S* is regarded as an agent to select relay $R_{n}$ and allocate power $P_{R_{n}}^{k}$ by learning with environment interactively.

Following, we formulate a RL framework for the joint relay selection and power allocation problem in EH-UASNs. In the formulated RL framework, there are 4 key factors to improve the learning efficiency of RL algorithm. They are shown as follows in detail.

**Agent**: The transmitting node *S*.

**State**: The setting of state is very important in RL. Given that node S only employs available state as learning information, reasonable state can make RL algorithm reveal the relationship between learning and environment preferably, thereby improving the performance of EH-UASNs.

Based on this idea, we proposed a more effective state expression (16) which adequately considers dynamic and unknown characteristics of EH-UASNs. The proposed state can be expressed as follows:

(16) $$\begin{array}{c} s^{k}=\left[B^{k},\alpha^{k},P_{n}^{k-1},G^{k-1}\right]. \end{array}$$

(1) Relay nodes battery, $B^{k}=\left[B_{1}^{k},B_{2}^{k},...,B_{n}^{k}\right]$, n∈N. Based on the above analysis, the battery of the relay nodes determines the upper limit of the power strategy, QoS and working life. In addition, unpredictable and stochastic EH is considered in the formulated problem. The remaining battery of relay nodes has the characteristics of dynamic and unpredictable, thereby impacting on the strategy selection of node *S* dynamically. Therefore, it is reasonable to set the battery of the relay nodes as a part of state, especially when EH is employed in UASNs.
(2) Current topology information of the network, $\alpha^{k}$, where $\alpha^{k}$ is a *N* dimensional vector encoded by one-hot. If the node $R_{n}$, n∈N is selected as the forward node at k−1 time slot, then $\alpha^{k}\left(n\right)=1$; otherwise, $\alpha^{k}\left(n\right)=0$. Topology information reflects the link status and relay forwarding link quality, which provides available learning information for node *S* to select relay in the future.
(3) Outdated transmitting power information of relay node, $P_{n}^{k-1}$. As an important parameter, the transmitting power $P_{n}^{k-1}$ is optimized to interact with underwater environment directly. In addition, the optimization objective is network performance of a working period, rather than that of instantaneous time. Historical optimization power information affects the action strategies of agent in the future, thereby affecting the cumulative performance in a working period. Therefore, it is necessary to take the outdated transmitting power of relay as a part of state.
(4) Outdated channel information, $G^{k-1}=\left[g_{SR_{n}}^{k-1},g_{R_{n}D}^{k-1},g_{SD}^{k-1}\right]$. The channel information characterizes the communication quality of link. Therefore, the joint strategies are directly impacted by the channel information. Due to the large underwater transmission delay, it is difficult to obtain the perfect real-time channel information. However, outdated channel information can be obtained by the well developed channel estimation methods. Although the historical outdated channel information is employed as a part of state, the RL algorithm can extract useful information to learn optimal strategies.

In References [27,28], the authors directly utilized the real-time channel information to build state. However, it is infeasible to obtain the real-time channel information due to high transmission delay in underwater environment. Unlike them, the proposed state expression employs the outdated channel information and outdated transmitting power to reveal the relationship between key factors. Moreover, the outdated information which is employed in the state expression is available in harsh underwater environment.

In this study, the agent selects an action *a* which consists of a relay selection strategy and a power allocation strategy to execute forwarding tasks. Therefore, when the agent selects an action, it means that the relay selection strategy and power allocation strategy are selected simultaneously. It is worth noting that the optimization objective is not only affected by the relay selection strategy but also by the power allocation strategy.

**Action**: The action is the joint strategies $R_{n}$ and $P_{R_{n}}^{k}$ which are optimized in this study. At time slot *k*, the node *S* selects an action $a^{k}=\left(R_{n},P_{R_{n}}^{k}\right)$ according to the observed state $s^{k}$. It is worth noting that the transmitting power of each candidate relay is discretized into 3 levels, and then the dimension of the action space is 3N.

**Reward**: The design of the reward function is crucial due to the fact that the reward function guides the agent to learn joint optimization strategies by interacting with underwater environment. In this study, we aim to design an effective reward function with the joint consideration of harvested energy and transmitting power as follows.

From (13)–(15), we can know that the higher transmitting power $P_{R_{n}}^{k}$ is, the greater capacity $C^{k}$ can be achieved. If more energy is harvested by a node, the battery of the node is more sufficient. Under the circumstances, higher power should be allocated to improve capacity of networks. However, due to the stochastic nature of EH, the harvested energy and remaining battery of nodes are unpredictable. In addition, given that the limited battery of the relay nodes, if the node keeps working with high transmitting power, its working life should be greatly reduced and outage probability of corresponding link should increase. Therefore, the reward function should guide the agent to maximize the cumulative capacity of network in a working period and reduce outages due to energy exhaustion by comprehensively considering harvested energy $E_{n}^{k-1}$ and instantaneous transmitting power of relay node $P_{R_{n}}^{k}$. Based on above analysis, on the premise of guaranteeing optimization objective, we aim to design a reward function which can balance instantaneous capacity $C^{k}$ and energy consumption $e_{n}^{k}$ to achieve long-term high-quality service. Under the reward function, the harvested energy should be adequately utilized to improve instantaneous capacity of EH-UASNs; the original battery is charily exploited to ensure the nodes have a certain amount of energy storage to avoid outages due to energy exhaustion, thereby improving working life and QoS in the future. Consequently, we proposed the reward function as follows:

rk=(17a)Ck−ωe−(PRnk−Enk−1),ifPRnk≤Bnk(17b)−ωe−(PRnk−Enk−1),else,

where ω is the penalty weight.

In (17a) and (17b), we proposed different reward forms in two cases. The details of analysis about the reward function are shown as follows:

(1) Non-outage case (Equation (17a))

In (Equation (17a)), $P_{R_{n}}^{k}\leq B_{n}^{k}$, It means that no outages happen on corresponding communication link. In this case, the proposed reward function consists of two items: the instantaneous capacity of network $C^{k}$ and the adaptive power penalty item $\omega e^{-(P_{R_{n}}^{k}-E_{n}^{k-1})}$.

The first item $C^{k}$ guides the agent to allocate resources effectively to maximize the instantaneous capacity. In addition, we add the second adaptive power penalty item $\omega e^{-(P_{R_{n}}^{k}-E_{n}^{k-1})}$ into the reward function to reduce long-term outage probability. The reward function which is specially constructed employs the properties of exponential function to adaptively utilize the harvested energy. If the transmitting power $P_{R_{n}}^{k}$ is less than the harvested energy $E_{n}^{k-1}$, it means the harvested energy $E_{n}^{k-1}$ is not fully utilized. Under the circumstances, given the proposed reward function of exponential form, the node *S* will obtain a relatively big penalty. The node *S* is encouraged to increase utilization of harvested energy in the future. Therefore, the harvested energy is adequately utilized to improve instantaneous channel capacity at the next time slot. If the transmitting power $P_{R_{n}}^{k}$ is greater than the harvested energy $E_{n}^{k-1}$, it means the harvested energy is fully utilized. Under the circumstances, the node *S* will obtain a relatively small penalty with the purpose of avoiding overuse of remaining battery, thereby improving the long-term service ability of nodes. Based on the the proposed reward function, the nodes can fully utilize harvested energy to improve instantaneous capacity. They can also retain remaining battery, thereby prolonging working life.

(2) Outage case (Equation (17b))

If the remaining battery $B_{n}^{k}$ is not enough to maintain the transmitting power $P_{R_{n}}^{k}$, which is $P_{R_{n}}^{k}>B_{n}^{k}$, an outage should happen on the corresponding communication link. And the instantaneous capacity $C^{k}=0$. Obviously, this result runs counter to our optimization objective. In this case, the reward is set to be a negative and the node *S* should obtain a very large penalty by the proposed reward form (Equation (17b)). Therefore, the node *S* will reasonably reduce the transmitting power $P_{R_{n}}^{k}$ according to the remaining battery of the relay nodes and the harvested energy to reduce the outage possibility in the future. In this case, The proposed reward form can prevent the relay nodes from overusing battery to improve instantaneous capacity, thereby reducing the outage probability and improving working life. In addition, The relay nodes can reserve a certain amount of batteries to respond to sudden service requests, thereby improving performance of entire network.

Based on above analysis, although the remaining battery of relay nodes is dynamic and unpredictable, the proposed reward function can make a tradeoff between instantaneous capacity and the long-term QoS to improve performance of EH-UASNs.

### 3.3. Deep RL-Based EH-Uasns Joint Resources Allocation Algorithm

In order to express our algorithm conveniently, the basic Q-learning algorithm which has been widely used in intelligent decision-making problems is shown firstly. In Q-learning algorithm, the agent converges its behavioral value function through continuous iterative learning. This behavioral value function is generally represented by Q-table to evaluate the expected discounted cumulative reward Q(s,a) after taking action *a* in a given state *s*, as follows:

(18) $$\begin{array}{c} Q_{\pi }\left(s,a\right)=E_{\pi }\left[\sum_{k=0}^{\infty }r^{k+1}+\gamma Q\left(s^{k+1},a^{k+1}\right)|s^{k}=s,a^{k}=a\right], \end{array}$$

where $r^{k+1}$ denotes the instant reward after action *a* is executed in the state *s*. γ(0<γ<1) is the discount factor; π is the strategy function that specifies the action π(s) that the agent will choose when in state *s*. After the strategy π(s) is executed, the Q-value is updated as follows:

(19) $$\begin{array}{c} Q_{update}\left(s^{k},a^{k}\right)=Q\left(s^{k},a^{k}\right)+\lambda \left(r^{k+1}+\gamma \max Q\left(s^{k+1},a^{k+1}\right)-Q\left(s^{k},a^{k}\right)\right), \end{array}$$

where λ(0<λ<1) is the learning rate.

In Q-table, the rows and columns represent the states and possible actions, respectively. After Q-learning converges, the optimal Q-table is learned. The optimal action *a* can be selected with the highest value of Q(s,a) in state *s*.

Q-learning algorithm shows high performance in small state space. However, when it is applied in larger state space, the performance of Q-learning would degenerate [32]. In complex dynamic EH-UASNs, the state space is very large and complex, which causes the dimension of the Q-table to become very large. It will require a lot of memory spaces and prolong the search time. It is very disadvantageous for underwater sensors whose energy and storage space are seriously limited. Moreover, some states are hard to explore, which makes it difficult for traditional Q-learning algorithm to find the global optimal strategy in EH-UASNs. The DQN algorithm which combines deep learning with reinforcement learning can achieve end-to-end learning from perception to action, thereby solving the bottleneck of Q-learning algorithm. And the DQN algorithm can deal with problems with almost infinite state space. Based on the above analysis, we proposed the JADQ-EH algorithm to cope with the time-varying and unpredictable underwater communication environment.

**Figure 3:**
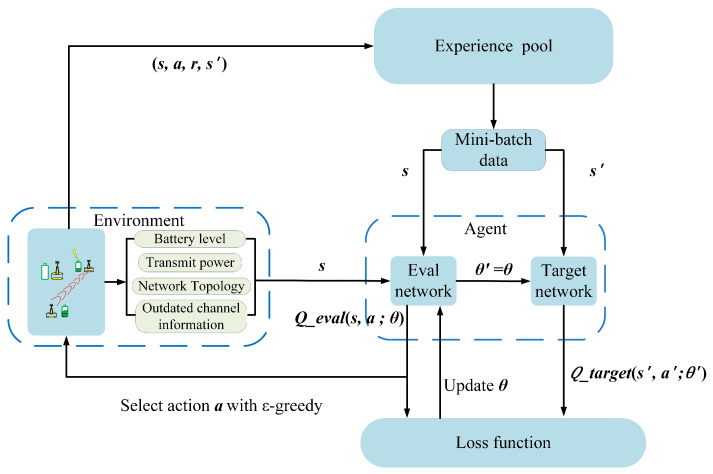
Framework of joint resource allocation based deep Q network algorithm considering energy harvesting (JADQ-EH) algorithm.

Unlike the Q-learning, the DQN algorithm does not need to establish an infinite Q-table to store Q-value. Meanwhile, it replaces the Q-table with deep neural networks which have shown the powerful ability in nonlinear fitting. Therefore, in DQN algorithm, the state space does not need to be discretized. The agent can search global optimal strategy by deepening exploration of the environment. The framework of the proposed JADQ-EH algorithm is shown in Figure 3.

The node *S* uses two independent neural networks, the Eval network with the current weight parameters θ and the Target network with the previous weight parameters $\theta^{\prime }$ as Q-value approximator. The structures of the two neural networks are exactly same, while the weight parameters are different. The neural network includes RNN and fully connected layers, hereinafter referred to as RFc which is employed in the proposed JADQ-EH algorithm. And the details of neural network will be shown later. The whole algorithm process is consisted of interactive experience collection stage and optimizing action strategy stage. Firstly, in the interactive experience collection stage, the node *S* collects experience by interacting with the environment and saves interactive data to experience pool which will be utilized to train RFc neural network in the second stage. The details are shown as follows: The node *S* obtains the state $s^{k}=\left[B^{k},\alpha^{k},P_{n}^{k-1},G^{k-1}\right]$ by observing the underwater communication environment and inputs it into the Eval network. The Eval network outputs the $Q_{-}eval\left(s,a;\theta \right)$ with the current weight parameters θ. And then, the agent selects an action $a^{k}=\left(R_{n},R_{R_{n}}^{k}\right)$ corresponding to the largest $Q_{-}eval$ value, consisting of relay selection strategy $R_{n}$ and power allocation strategy $P_{R_{n}}^{k}$, to perform the forwarding task. The node *S* also explores a random action by ε−greedy scheme [27]. ε is the probability of randomly selecting actions, as follows:

(20) $$\begin{array}{c} \varepsilon^{k}=\left\{\begin{array}{cc} 1-0.001\times \;\mathrm{episode}, & \varepsilon^{k}<0.9\\ 1-0.9, & \mathrm{else} \end{array}\right., \end{array}$$

where the episode is the number of training.

After the action $a^{k}$ is executed, the environment transmits to the next state $s^{k+1}$. Then, the agent obtains the feedback from the environment $C^{k}$, $E_{n}^{k-1}$ and obtains the reward $r^{k+1}$ by Equation (17). In each time slot *k*, the node *S* stores its interactive experience tuple $\left(s^{k},a^{k},r^{k+1},s^{k+1}\right)$ into the experience pool with storage capacity H.

Following, we show the details of the optimizing action strategy stage. Given that our optimization objective is to maximize cumulative capacity in *K* continuous working time slots, the node *S* trains the RFc every *K* time slots. In the process of training, the mini-batch data is randomly selected from the experience pool to update the weight parameters θ of Eval network. The RFc weight parameters θ are updated by minimizing the loss function Equation (21) with the RMSPropOptimizer method.

(21) $$\begin{array}{c} \mathrm{Loss}\left(\theta \right)=E\left[{\left(y-Q_{-}eval\left(s,a;\theta \right)\right)}^{2}\right]. \end{array}$$

The target Q-value *y* is given as follows

(22) $$\begin{array}{c} y=r+\gamma \max_{a^{\prime }}Q_{-}\mathrm{target}\left(s^{\prime },a^{\prime };\theta^{\prime }\right). \end{array}$$

The evaluate Q-value is the output of the Eval network with weight parameters θ. More specifically, the new RFc weight parameters are updated by (23),

(23) $$\begin{array}{c} \begin{array}{cc} \theta_{update} & =\theta +\nabla_{\theta }Loss\left(\theta \right)\\ =E\left[\left(r+\gamma \max_{a^{\prime }}Q_{-}target\left(s^{\prime },a^{\prime };\theta^{\prime }\right)\right.\right. & \left.\left.-Q_{-}eval\left(s,a;\theta \right)\right)\nabla_{\theta }Q_{-}eval\left(s,a;\theta \right)\right], \end{array} \end{array}$$

where the $\nabla_{\theta }Q_{-}eval\left(s,a;\theta \right)$ is the gradient of θ. The node *S* replaces the weight parameters $\theta^{\prime }$ with θ every 500 episodes.

Due to the large delay of underwater transmission, it is hard to obtain the perfect channel information in time. Given the cumulative learning and information reasoning capabilities of RNN, the historical outdated channel information is available for RNN to learn. In addition, given that the strongly time-varying channel information and EH process, traditional algorithms are more difficult to solve the proposed optimization problem. However, the RNN has inherent advantages in extracting useful information from data with time series characteristics. Consequently, the RFc neural network is adopted to improve the learning ability of node *S*.

**Figure 4:**
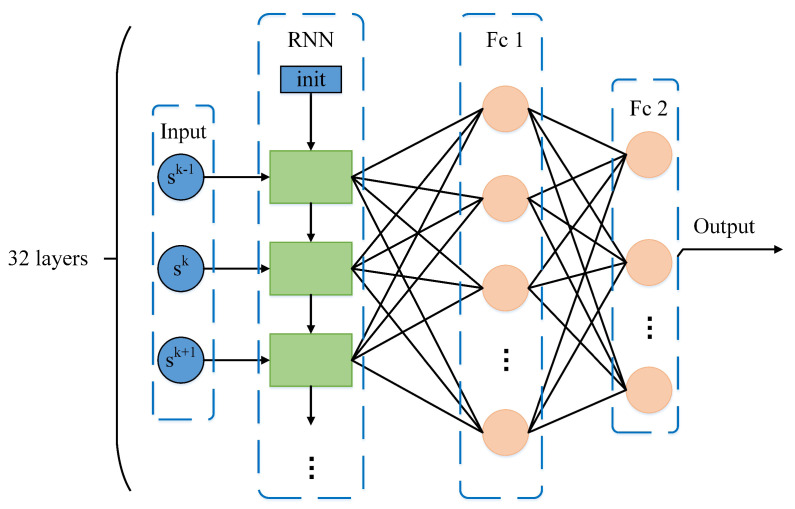
Structure of RFc neural network for approximating Q-function.

Further, Figure 4 shows the detailed structure of the RFc. In Figure 4, the RFc includes 32 RNN Cells and 2 fully connected (Fc) layers. The input of the RFc is $s^{k}$. The hidden layer and output of RNN part employ Tanh and ReLU as activation functions, respectively. The first Fc layer has 50 ReLUs, and the second Fc layer has 3N outputs. The trick of soft update can prevent gradient disappearance and gradient explosion in training. It has been widely utilized in training RNN networks. In this study, the parameters $\theta^{\prime }$ are updated by

(24) $$\begin{array}{c} \theta^{\prime }=\beta \theta^{\prime }+\left(1-\beta \right)\theta , \end{array}$$

where the β is the soft update weight.

Based on the given descriptions and analysis, the pseudocode of JADQ-EH algorithm is given as follows in Algorithm 1:
**Algorithm 1** The proposed JADQ-EH algorithmInitialize $B_{max}$, $P_{S}$, Φ; Initialize the learning rate λ, the discount factor γ, Eval network weight parameters θ, and Target network weight parameters $\theta^{\prime }$, the *K*, H=∅, $\varepsilon^{0}$, minibatch; **for** episode = 1,2,3,..., **do**
    **for** *k*=1,2,3,...,*K* **do**          input state $s^{k}=\left[B^{k},\alpha^{k},P_{n}^{k-1},G^{k-1}\right]$ into the Eval network and obtain $Q_{-}eval$;         choose action $a^{k}$ with ε−greedy;         execute $a^{k}$ and obtain the reward $r^{k+1}$, $s^{k+1}$;         save experience $\left(s^{k},a^{k},r^{k+1},s^{k+1}\right)$ to experience pool;     **end for**     sample a minibatch data from experience pool;     train the Neural networks using the minibatch data with the loss function (21);      update Eval network weight parameters θ via (23);     **if** episode mod M == 0 **then**
        replace Target network weight parameters $\theta^{\prime }$ with θ via (24);     **end if** **end for**

The proposed JADQ-EH algorithm consists of two stages: The interactive experience collection stage is shown in 4th–9th statements; The optimizing action strategy stage is shown in 10th–15th statements. The relevant analysis have been shown above and the details will not be discussed here to avoid repetition.

**Remark 1.** *(Outdated information): From the perspective of long-time service, the cumulative capacity maximization of a working period is our optimization objective, rather than the instantaneous capacity. In the proposed JADQ-EH algorithm, the available historical outdated information is utilized to improve the cumulative capacity and QoS. Therefore, we employ the outdated information $P^{k-1}$, $G^{k-1}$ as a part of state and fully utilize the outdated information to improve learning ability. In the complex underwater environment, real-time channel information $G^{k}$ is hard to be obtained. However, outdated information $G^{k-1}$ can be easily obtained. In addition, $P^{k-1}$ is the local information of nodes. Consequently, the outdated information $P^{k-1}$, $G^{k-1}$ which the agent requires in the proposed JADQ-EH algorithm can be easily obtained.*

**Remark 2.** *(Energy harvesting): Given that the dynamic energy harvesting is considered in the proposed problem, the joint optimization problem is more stochastic and harder to solve. In the proposed JADQ-EH algorithm, the proposed adaptive power penalty term can guide the node S to adjust the action strategy by utilizing the gap between allocated power and harvested energy. The harvested energy is fully utilized to improve instantaneous capacity and the original energy of relay nodes is charily utilized to ensure the nodes have a certain amount of energy storage to avoid outages in the future. Thus, the working life of nodes is extended and long-term QoS is improved. Essentially, the proposed reward function comprehensively considers the battery trend and transmitting power of nodes to balance instantaneous energy utilization and long-term QoS. Consequently, the proposed JADQ-EH algorithm can efficiently solve the complex joint optimization problem.*

**Remark 3.** *(Computational complexity): The computational complexity of the proposed JADQ-EH algorithm depends on the RFc network. The computational complexity of RNN network is $O_{1}=\left(\right|$s${|*\left|RNN\;Cells\right|}^{2})$[33], where |⋅| represents the number of elements. The computational complexity of the first Fc layer is $O_{2}=\left(50*\right|$s|). The computational complexity of the second Fc layer is $O_{3}=\left(50*3N*\right|$s|). Consequently, the computational complexity of the JADQ-EH algorithm is $O_{1}$+$O_{1}$+$O_{1}$=(|s${|*\left|RNN\;Cells\right|}^{2}+50*|$s|+50∗3N∗|s|).*

## 4. Simulation Results and Analysis

### 4.1. Simulation Setting

We consider two simulation scenarios of the UASNs system. The simulation scenario 1 consists of 1 transmitting node, 3 relay nodes ($R_{0}$, $R_{1}$, $R_{2}$) and 1 desired receiving node in a 200 m × 200 m communication area. The transmitting node at (0, 0) m broadcasts signal to relay nodes and desired receiving node with transmitting power $P_{S}=1$ mW. The relay nodes are dropped randomly in the communication area. And the desired receiving node at (200, 200) m receives the signal and forwards to base station [27]. We further introduce a simulation scenario 2 which consists of 7 nodes. 1 transmitting node, 5 relay nodes ($R_{0}$, $R_{1}$, $R_{2}$, $R_{3}$, $R_{4}$) and 1 desired receiving node are included in the scenario 2. It is worth noting that the topology of UASN is refreshed with random mechanism every episode, which increases the dynamicity and unpredictability. The probability of randomly choosing actions $\varepsilon^{0}$ = 0.001. The other specific parameters are given in Table 2.

### 4.2. Results and Analysis

In this section, we compare the performance of the proposed JADQ-EH algorithm with other three algorithms: (i) Greedy optimal joint resources allocation (GOA) algorithm which assumes the perfect channel information is known. It is worth noting that the GOA algorithm calculates the corresponding network capacity of all links with the premise of no outages. And it selects the link which can maximize its capacity as the optimal strategy; (ii) Random algorithm; (iii) the basic Q-learning algorithm. To verify the effectiveness of the proposed algorithm, we evaluate the performance in terms of $C_{total}$; the number of outages in a working period $O_{total}$; the average working lifetime $J_{ave}$ and the remaining battery at the end of every working period $\hat{B}$.

**Figure 5:**
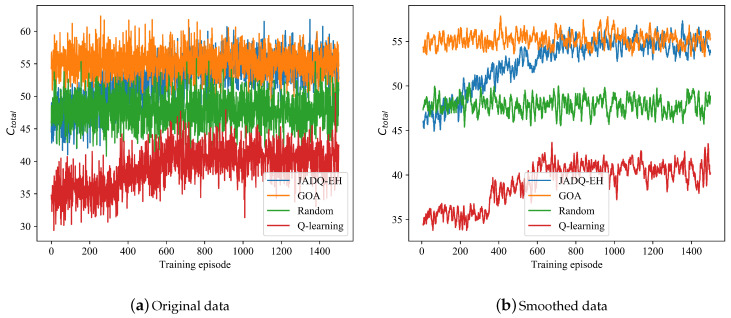
$C_{total}$ comparison of different algorithms under scenario 1.

Figure 5 shows the $C_{total}$ optimization processes of JADQ-EH algorithm and other algorithms under scenario 1. Given that the curves are too dense based on original data in Figure 5a, the curves are smoothly processed to show the trend more clearly in Figure 5b. It is worth noting that the smoothed data is the average value of a rolling window which consists of 8 original data. First, based on Figure 5, the analysis of comparison between JADQ-EH algorithm and GOA algorithm are shown as follows. In initial training phase, $C_{total}$ obtained by the GOA algorithm is superior that obtained by the proposed JADQ-EH algorithm. The main reason is because the GOA algorithm assumes the real-time perfect system information is known. In other words, at any moment in the entire process, GOA algorithm can obtain perfect channel information timely. Therefore, the GOA algorithm can achieve optimal joint relay selection and power allocation strategies. However, it is infeasible to obtain perfect real-time channel information in harsh underwater environment. With the continuous exploration and learning of the environment, the proposed JADQ-EH algorithm achieves the optimal performance (performance of GOA algorithm) without any prior channel information. Moreover, it is worth noting that the partial learning information of the proposed JADQ-EH algorithm is historical information which can be obtained easily. In addition, we set outdated channel information as a part of state, rather than any prior information about channel. These verify the reliability and rationality of the designed state expression and the learning ability of the proposed JADQ-EH algorithm in harsh underwater environment.

From Figure 5, we can know that the proposed JADQ-EH algorithm absolutely outperforms the other algorithms. In Figure 5, the performance obtained by JADQ-EH algorithm is almost the same as that obtained by Random algorithm, before about 200 episodes. This is mainly because that the agent randomly chooses actions to deepen environment exploration in initial training phase of the proposed JADQ-EH algorithm. However, the proposed JADQ-EH algorithm obviously outperforms the Random algorithm after 200 episodes. The learning ability of the proposed JADQ-EH algorithm is verified. In addition, the basic Q-learning algorithm shows the worst performance in the proposed joint optimization problem. The main reason is because the state space of the proposed problem is almost infinite. The agent can not explore all states with finite Q-table in basic Q-learning algorithm. Therefore, the agent falls into local optimal strategy under basic Q-learning algorithm. However, the proposed JADQ-EH algorithm utilizes RFc network to deal with infinite state space which overcomes the bottleneck of Q-learning algorithm. Simulation results verify the proposed effective state expression which reveals the environment characteristics can improve learning ability.

Figure 5 only shows the optimization process. In Table 3, we further show the average cumulative capacity in a working period $C_{total}^{ave}$ comparison of different algorithms after convergence under scenario 1. It clearly shows that the proposed JADQ-EH algorithm has superior performance in the final convergence phase. The results in Table 3 are consistent with the curves in Figure 5. These further verify the above analysis and the superiority of the proposed JADQ-EH algorithm.

**Figure 6:**
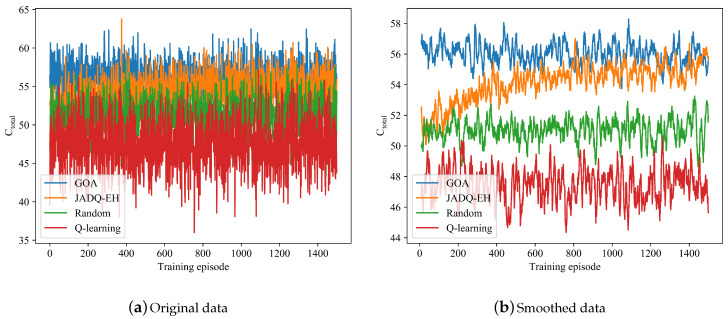
$C_{total}$ comparison of different algorithms under scenario 2.

Different from scenario 1, Figure 6 shows that the Q-learning algorithm does not achieve convergence under scenario 2. The main reason is because the state space is quite large and complex under scenario 2 which has more candidate relay nodes and stronger randomness. Given that the Q-learning algorithm cannot tackle large state space, the Q-learning algorithm does not learn optimization strategy knowledge under scenario 2. With the continuous exploration and learning of the environment, $C_{total}$ achieved by the proposed JADQ-EH algorithm is close to that achieved by the GOA algorithm. The features of $C_{total}$ under scenario 2 are similar to these of Figure 5 under scenario 1. Given that the reasons of the features have been shown in the analysis of scenario 1, we do not repeat it here.

Table 4 shows that $C_{total}^{ave}$ achieved by the proposed JADQ-EH algorithm is superior to these achieved by Random algorithm and Q-learning algorithm under scenario 2. Although the capacity achieved by JADQ-EH algorithm show a little superiority in Table 4, the cumulative services (outage, working lifetime) achieved by JADQ-EH algorithm absolutely outperform other algorithms. The other algorithms only consider the instantaneous capacity and ignore the long-term QoS. Outages which are caused by energy exhaustion easily appear under other algorithms. The outage performance and working life are sacrificed. However, the proposed JADQ-EH algorithm jointly consider the instantaneous capacity and the efficient utilization of energy. The proposed JADQ-EH algorithm not only maximizes capacity but also minimizes number of outages. The following simulation results about $O_{total}$ and $O_{total}^{ave}$ can verify the above analysis.

**Figure 7:**
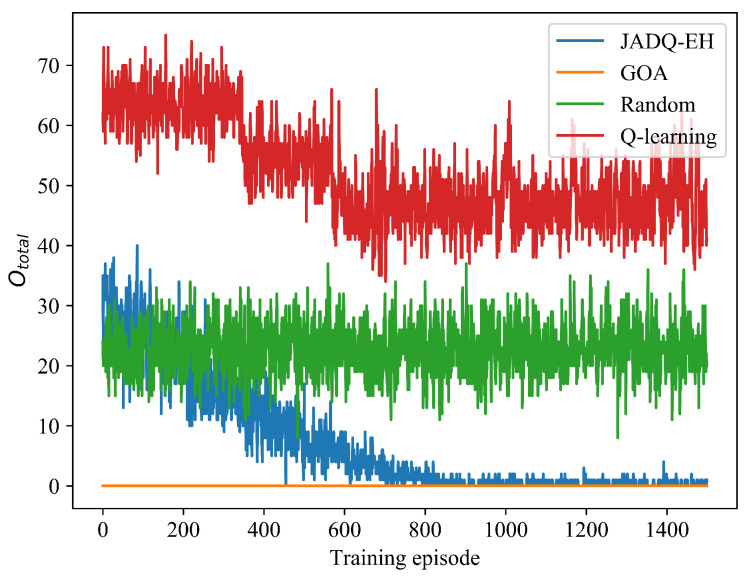
$O_{total}$ comparison of different algorithms under scenario 1.

The QoS is not only affected by capacity but also outage. When the battery of relay nodes can not afford the allocated transmitting power, an outage will happen on corresponding communication link. In a working period, too many outages will cause the severe degradation. Figure 7 shows $O_{total}$ comparison of different algorithms under scenario 1. Given that the GOA algorithm selects an action under the condition of no outages, $O_{total}$ = 0 in the whole process. In Figure 7, with the learning time increasing, $O_{total}$ achieved by proposed JADQ-EH algorithm is declining. The performance obtained by the proposed JADQ-EH algorithm is almost the same as that obtained by GOA algorithm after 800 episodes. The main reason is because the proposed reward function can comprehensively balance the energy utilization and optimization objective to improve QoS. If the links generate outages, the agent will obtain a big penalty. Therefore, the agent will try to avoid outages with the guidance of the proposed reward function in the process of learning. In addition, the proposed JADQ-EH algorithm absolutely outperforms the other algorithms. The main reason is because the agent can obtain more training samples to explore and learn the dynamic EH-UASNs to improve QoS as the training episodes increase. The Random algorithm always makes decisions randomly. Therefore, the $O_{total}$ achieved by Random algorithm has no obvious change. Given that the Q-learning is not applicable for large state space, it shows the worst performance in EH-UASNs. Figure 7 shows that the starting points of the four resource allocation algorithms are inconsistent. The main reasons are that the proposed JADQ-EH algorithm collects the interactive experience first, and then optimize learning at the end of each working period. Therefore, the curve achieved by the JADQ-EH algorithm is similar to that of Random algorithm at the starting point. However, the learning mode of Q-learning algorithm is one-step update mode. The limited learning ability of Q-learning algorithm cannot support the agent to learn better strategy after several learning times. Therefore, the $O_{total}$ of the Q-learning algorithm is higher at the starting point. Given that the GOA selects an action under the condition of no outages, $O_{total}$ = 0 in the whole process.

Table 5 shows the average number of outages in a working period $O_{total}^{ave}$ comparison of different algorithms after convergence under scenario 1. It clearly shows that $O_{total}^{ave}$ achieved by the proposed JADQ-EH algorithm is close to 0. These verify the advantages of the proposed JADQ-EH algorithm in avoiding outages and improving QoS. Consequently, the proposed JADQ-EH algorithm shows the high applicability in dynamic unpredictable EH-UASNs.

**Figure 8:**
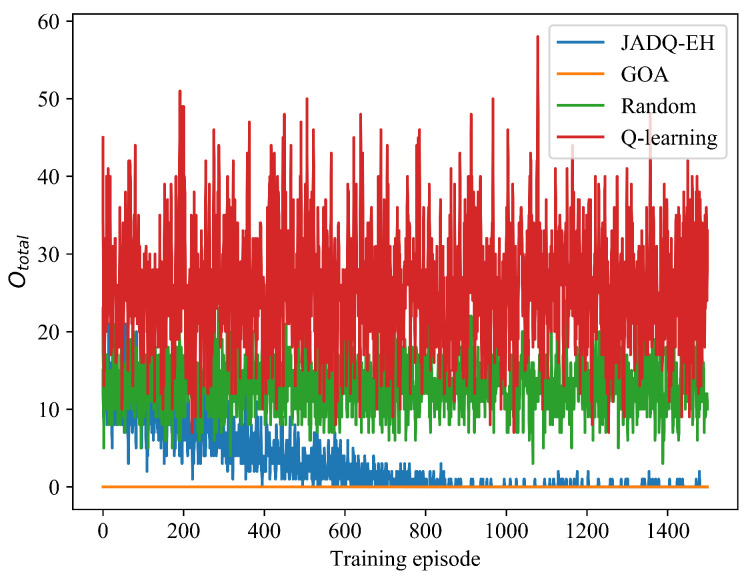
$O_{total}$ comparison of different algorithms under scenario 2.

Figure 8 shows the $O_{total}$ optimization processes of JADQ-EH algorithm and other algorithms under scenario 2. Table 6 shows the $O_{total}^{ave}$ comparison of different algorithms under scenario 2. Figure 8 and Table 6 show that the features of curves trend under scenario 2 are similar to that of scenario 1. Figure 8 and Table 6 clearly show that the proposed JADQ-EH algorithm absolutely outperforms the Random and Q-learning algorithms in minimizing $O_{total}$. The outage probability achieved by the JADQ-EH algorithm is only 0.07% under scenario 2. The $O_{total}^{ave}$ achieved by Random algorithm is 106 times that achieved by JADQ-EH algorithm. These further verify the universal applicability of the proposed JADQ-EH algorithm.

**Figure 9:**
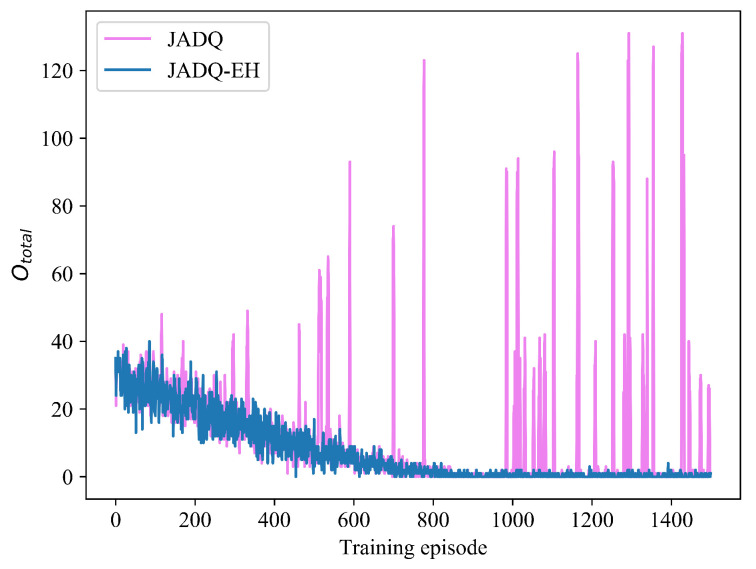
$O_{total}$ comparison of JADQ-EH and JADQ under scenario 1.

In order to verify the efficiency of proposed JADQ-EH algorithm further, the performance achieved by the proposed JADQ-EH algorithm is compared with that achieved by the JADQ algorithm which does not consider EH in reward function. Figure 9 shows the $O_{total}$ comparison between JADQ-EH algorithm and JADQ algorithm under scenario 1. The Table 7 shows the $O_{total}^{ave}$ comparison between JADQ-EH algorithm and JADQ algorithm after convergence under scenario 1. In Figure 9, convergence trend of $O_{total}$ in JADQ algorithm is similar to that in JADQ-EH algorithm. However, numerous points where $O_{total}$ increases sharply are shown on the curve of JADQ algorithm, while the JADQ-EH algorithm does not. Table 7 shows that the $O_{total}^{ave}$ achieved by JADQ algorithm is 17.6 times that achieved by JADQ-EH algorithm after convergence. In the proposed JADQ-EH algorithm, the $O_{total}^{ave}$ is only 0.396. The main reason is because the proposed reward function in the JADQ-EH algorithm not only guarantees the optimization objective of maximizing capacity, but also considers energy utilization to reduce outages in dynamic and unknown EH-UASNs. The constructed reward function guides the agent to adequately utilize the harvested energy and charily to exploit the original battery by employing the adaptive power penalty item. The relay nodes can be prevented from overusing the battery and reserve a certain amount of battery to cope with the sudden service requests, thereby reducing $O_{total}$.

**Figure 10:**
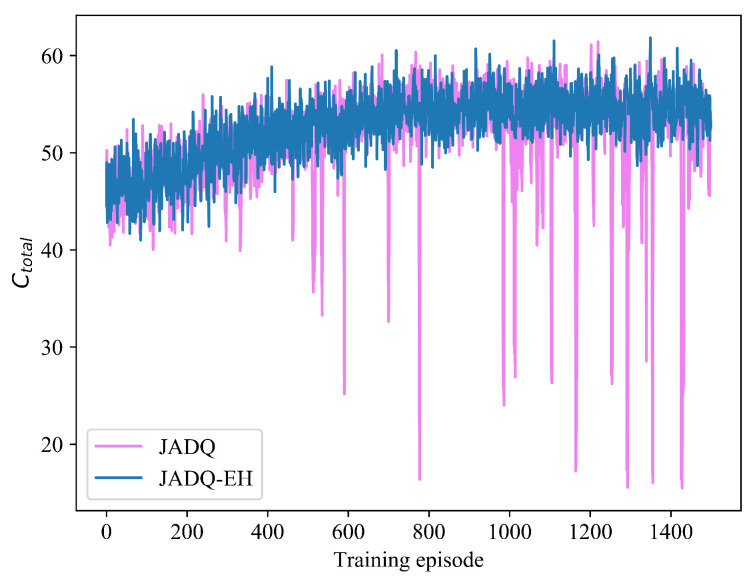
$C_{total}$ comparison of JADQ-EH and JADQ under scenario 1.

Figure 10 shows the $C_{total}$ comparison between JADQ-EH algorithm and JADQ algorithm under scenario 1. Table 8 shows the $C_{total}^{ave}$ comparison between JADQ-EH algorithm and JADQ algorithm after convergence under scenario 1. From Figure 10, we can know that $C_{total}$ obtained by the proposed JADQ-EH algorithm is superior to that obtained by JADQ algorithm. Table 8 further verifies the result. In addition, numerous points where the $C_{total}$ drops sharply can be observed on the curve of JADQ algorithm in the whole process. The main reason is because the JADQ algorithm only considers to meet the $C_{total}$ and does not care remaining energy status. Therefore, the $C_{total}$ and QoS achieved by the JADQ algorithm are degraded due to energy exhaustion. In Table 7 and Table 8, $O_{total}^{ave}$ achieved by JADQ is 17.6 times that achieved by JADQ-EH algorithm while $C_{total}^{ave}$ obtained by JADQ-EH algorithm is higher than that obtained by JADQ algorithm. It means that the proposed reward function can not only reduce outages but also improve $C_{total}$ and QoS. Consequently, the proposed JADQ-EH algorithm has strong learning and optimization ability to deal with dynamic and complex EH-UASNs.

**Figure 11:**
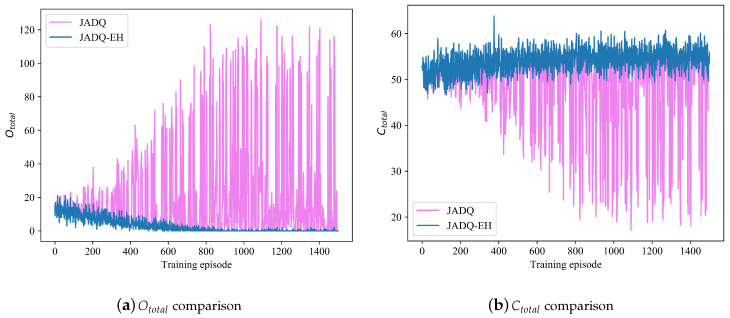
Performance comparison between JADQ-EH and JADQ about $O_{total}$ and $C_{total}$ under scenario 2.

Figure 11a,b show the performance comparison between JADQ-EH algorithm and JADQ algorithm about $O_{total}$ and $C_{total}$ under scenario 2. The JADQ-EH and JADQ algorithms under scenario 2 show the similar convergent tendency about $O_{total}$ and $C_{total}$ to that under scenario 1, respectively. However, Figure 11a shows that more points where $O_{total}$ increases sharply are shown on the curve of JADQ algorithm under scenario 2 than the curve of scenario 1, while the JADQ-EH algorithm does not. Accordingly, the similar phenomenon occurs in Figure 11b. This is mainly because that the energy condition and channel situation are more complex and unpredictable under scenario 2, thereby enlarging the state space strongly. The JADQ algorithm, which does not consider adaptive power penalty in reward function, is more difficult to tackle the complex and dynamic scenario 2. Therefore, the performance achieved by JADQ algorithm is severely degraded under scenario 2 than that under scenario 1.

Table 9 shows the performance comparison between JADQ-EH and JADQ about $O_{total}^{ave}$ and $C_{total}^{ave}$ under scenario 2. It clearly shows that the proposed JADQ-EH algorithm absolutely outperforms the JADQ algorithm. Table 9 shows that $O_{total}^{ave}$ achieved by JADQ is 224 times that achieved by JADQ-EH. These verify the superiority of the proposed JADQ-EH algorithm under the scenario 2. The universal applicability of the proposed JADQ-EH algorithm is further verified.

We analyze the working lifetime of each relay node under scenarios 1 and 2. We define the time when the first outage occurs in each working period after algorithm convergence as the working lifetime *J*. We employ the average working lifetime $J_{ave}$ to verify the universal applicability of the proposed JADQ-EH algorithm.

Table 10 shows that $J_{ave}$ of relay nodes under JADQ-EH algorithm absolutely outperform the other algorithms under any scenarios. Taking the $R_{1}$ and $R_{2}$ for example, $J_{ave}$ achieved by the proposed JADQ-EH algorithm can reach at least 83% of the working period. However, $J_{ave}$ achieved by Random and Q-learning are at most 15% and 7.6% of the working period, respectively. The superiority of the proposed JADQ-EH algorithm in improving the working lifetime is verified.

Based on the above analysis, we can know that $C_{total}$ and $O_{total}$ obtained by the proposed JADQ-EH algorithm are close to optimal performance (the performance achieved by GOA algorithm). In order to verify the superiority of the proposed JADQ-EH algorithm in improving long-term service capability, we evaluate the performance about remaining battery of relay node at the end of every working period $\hat{B}$ under scenario 1.

**Figure 12:**
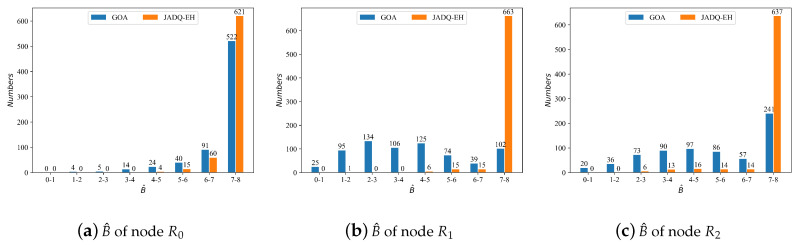
$\hat{B}$ of each node comparison between Greedy optimal joint resources allocation (GOA) and JADQ-EH under scenario 1.

Figure 12 shows the $\hat{B}$ statistical information of each node using JADQ-EH algorithm and GOA algorithm under scenario 1. The x-axis and y-axis indicate different battery levels and the corresponding number of battery levels, respectively. The maximum battery capacity $B_{max}$ of each relay node is 8 mJ. Figure 12 shows that $\hat{B}$ of each relay node almost stay above 4 under the proposed JADQ-EH algorithm. It means that each node can retain at least half of $B_{max}$ at the end of every working period. On the other words, each node can store enough energy to improve the long-term service capability. However, the $\hat{B}$ distribution of each node achieved by the GOA algorithm is relatively scattered. It means that number of battery levels which are at lower battery levels is more. Therefore, sudden service requests may not be served under GOA algorithm. Especially, the battery levels mainly concentrate in 7–8 mJ under the JADQ-EH algorithm. The relay nodes can tackle sudden and continuous requests with enough battery under the JADQ-EH algorithm. The main reason is because the proposed adaptive power penalty item guides the agent to adequately utilize the harvested energy and charily to exploit the original battery in the proposed JADQ-EH algorithm. The relay nodes can be prevented from overusing the battery and reserve as much battery as possible. The superiority of the proposed JADQ-EH algorithm in improving the long-term service capability is verified.

## 5. Conclusions

In this study, a cooperative transmission joint strategy optimization problem for dynamic unpredictable EH-UASNs is proposed, which maximizes the cumulative capacity in a working period. The joint relay selection and power allocation problem for EH-UASNs is formulated as a MDP without the knowledge of environment model. We propose a more effective state expression which does not require any prior channel information to provide available abundant learning information. It can better depict the rule of interaction between environment and learning. A reward function is further proposed to lead nodes to adaptively utilize harvested energy by employing the adaptive power penalty item. In this way, the proposed reward function can make a trade-off between instantaneous energy consumption and remaining energy. Benefits by the proposed state expression and reward function, the proposed JADQ-EH algorithm can balance the instantaneous capacity and long-term QoS to improve cumulative service ability of networks. The proposed learning algorithm can optimize the joint strategies by efficient interactive learning with environment without any prior information. Therefore, the proposed JADQ-EH algorithm shows strong applicability in the dynamic complex EH-UASNs with difficult-to obtain perfect real-time channel information. Finally, with the comparative analysis of simulation results, we verify that the proposed JADQ-EH algorithm can reduce the outage probability, improve the cumulative network capacity performance and long-term service ability.

## Figures and Tables

**Table 1:** Literature overview.

Existing Studies	Prior CSI	Environment Model	EH	Joint Problem
[8,27]	✔	✔	✗	✗
[17,21]	✔	✔	✔	✗
[22]	✗	✗	✗	✗
[15,16]	✔	✔	✔	✔
Our Work	✗	✗	✔	✔

**Table 2:** Simulation parameters.

Network Model Parameters		
N	Number of relay nodes	3/5
*K*	Continuous working period (s)	180
$P_{S}$	Transmitting power of node *S* (mW)	1
$P_{R_{0}}$	Transmission power of relay node $R_{0}$ (mW)	[1, 1.5, 2]
$P_{R_{1}}$	Transmission power of relay node $R_{1}$ (mW)	[3, 3.5, 5]
$P_{R_{2}}$	Transmission power of relay node $R_{2}$ (mW)	[5, 5.5, 6]
$P_{R_{3}}$	Transmission power of relay node $R_{3}$ (mW)	[1.2, 1.7, 2.2]
$P_{R_{4}}$	Transmission power of relay node $R_{4}$ (mW)	[3.2, 3.7, 4.2]
**Energy & Problem Parameters**		
$B_{max}$	Maximum battery capacity of each relay node (mJ)	8
$E_{n}^{k-1}$	Harvested energy by relay node $R_{n}$ in time slot k−1 (mJ)	[0, 3]
$e_{n}^{k}$	Energy consumed by the relay node $R_{n}$ in time slot *k* (mJ)	∖
$B_{n}^{k}$	Battery of relay node $R_{n}$ at the beginning of time slot *k* (mJ)	∖
$P_{R_{n}}^{k}$	Transmission power of node Rn in the time slot *k* (mW)	∖
$P_{n,max}$	Maximum transmitting power of relay $R_{n}$ (mW)	∖
**Training Parameters**		
λ	Learning rate	0.9
β	Soft update weight	0.5
γ	Discount factor	0.9
ω	Penalty weight	0.5
episode	Number of training	1500
minibatch	Number of sampling	32
H	Storage capacity of experience pool	5000
**Evaluation Indicators**		
$C_{total}$	Cumulative capacity in a working period	∖
$O_{total}$	Number of outages in a working period	∖
$C_{total}^{ave}$	Average cumulative capacity in a working period after convergence	∖
$O_{total}^{ave}$	Average number of outages in a working period after convergence	∖
$J_{ave}$	Average working lifetime	∖
$\hat{B}$	Remaining battery at the end of every working period after convergence (mJ)	∖

**Table 3:** $C_{total}^{ave}$ comparison of different algorithms under scenario 1.

Algorithm	JADQ-EH	GOA	Random	Q-Learning
$C_{total}^{ave}$	54.688	55.241	47.834	40.602

**Table 4:** $C_{total}^{ave}$ comparison of different algorithms under scenario 2.

Algorithm	JADQ-EH	GOA	Random	Q-Learning
$C_{total}^{ave}$	54.851	56.0201	51.048	47.455

**Table 5:** $O_{total}^{ave}$ comparison of different algorithms under scenario 1.

Algorithm	JADQ-EH	GOA	Random	Q-Learning
$O_{total}^{ave}$	0.396	0	22.776	47.003

**Table 6:** $O_{total}^{ave}$ comparison of different algorithms under scenario 2.

Algorithm	JADQ-EH	GOA	Random	Q-Learning
$O_{total}^{ave}$	0.124	0	13.15	25.490

**Table 7:** $O_{total}^{ave}$ comparison of JADQ-EH and JADQ under scenario 1.

Algorithm	JADQ-EH	JADQ
$O_{total}^{ave}$	0.396	6.947

**Table 8:** $C_{total}^{ave}$ comparison of JADQ-EH and JADQ under scenario 1.

Algorithm	JADQ-EH	JADQ
$C_{total}^{ave}$	54.688	52.709

**Table 9:** Performance comparison between JADQ-EH and JADQ about $O_{total}^{ave}$ and $C_{total}^{ave}$ under scenario 2.

Algorithm	JADQ-EH	JADQ
$O_{total}^{ave}$	0.124	27.864
$C_{total}^{ave}$	54.851	46.520

**Table 10:** The $J_{ave}$ of relay nodes comparison under different scenarios.

Simulation Scenario	Relay	JADQ-EH	Random	Q-Learning
Scenario 1	$R_{0}$	180 s	180 s	180 s
	$R_{1}$	179.84 s	26.63 s	13.82 s
	$R_{2}$	149.78 s	16.03 s	5.41 s
Scenario 2	$R_{0}$	180 s	180 s	180 s
	$R_{1}$	179.88 s	95.11 s	63.576 s
	$R_{2}$	169.98 s	18.82 s	15.92 s
	$R_{3}$	180 s	179.79 s	180 s
	$R_{4}$	179.42 s	151.17 s	52.91 s
